# New onset Behçet's disease in repeat pregnancy

**DOI:** 10.1016/j.jdcr.2024.10.040

**Published:** 2025-01-29

**Authors:** Lillian Xie, Travis Benson, Eric E. Morgan, William Lewis, Jonathan J. Kotzin, Ethan Craig, Misha Rosenbach

**Affiliations:** a Department of Dermatology, Hospital of the University of Pennsylvania, Philadelphia, Pennsylvania; b Department of Pathology and Laboratory Medicine, Hospital of the University of Pennsylvania, Philadelphia, Pennsylvania; c Department of Rheumatology, Hospital of the University of Pennsylvania, Philadelphia, Pennsylvania

**Keywords:** autoimmune, Behçet's disease, pregnancy, vasculitis

## Introduction

Behçet's disease (BD) is a rare, autoimmune systemic vasculitis that is classically associated with oral and genital ulcerations, recurrent uveitis, and erythema nodosum-like lesions. BD most commonly affects women of childbearing age, and several studies have examined its relationship with pregnancy. During pregnancy, treatment decisions must weigh impacts on both the mother and developing fetus. We report a case of BD with initial presentation during a repeat pregnancy.

## Case report

An Uzbekistani woman in her 20s G2P1001 at 35 weeks presented with 10 days of yellow-green vaginal discharge and 4 days of vaginal and oral aphthae, shortness of breath, pleuritic chest pain, and tender nodules on her extremities. She had no other medical problems and took no medications aside from a daily prenatal vitamin. Two years prior to the initial consultation, the patient had an uncomplicated vaginal delivery in South Korea. She reported a history of recurrent painful oral lesions along her buccal mucosa and inner lip for the past 5 years but no prior genital lesions.

**Fig 1:**
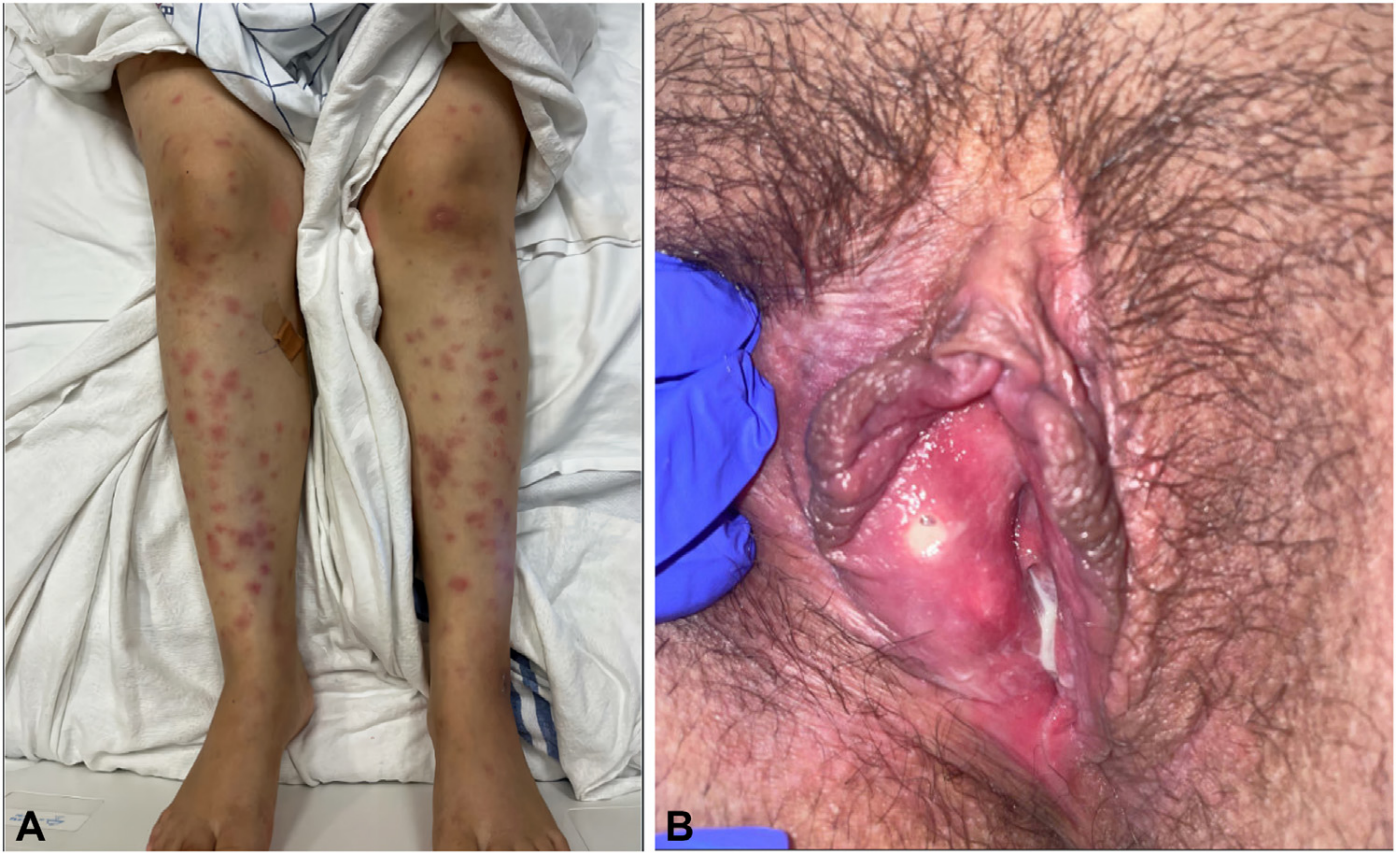
Diffuse nonblanching intense erythematous papules and nodules on the legs (**A**). Ulcer on right medial labia (**B**).

Physical examination was significant for erythematous, tender nodules on the shins, bilateral wrists, elbows, and multiple acneiform lesions on her upper back and abdomen. Notably, she had a single punched-out ulcer on her right medial labium minus, an ulcer on her buccal mucosa, and geographic tongue (Fig 1). Initial workup ruled out acute coronary syndrome with negative troponins and sinus tachycardia on EKG. Chest CT demonstrated no pulmonary embolism or hilar adenopathy. An echocardiogram showed a small loculated pericardial effusion. An extensive infectious workup, including tests for tuberculosis and group A streptococcus, was unrevealing. Subsequent HLA phenotyping was positive for B:13,51.

**Fig 2:**
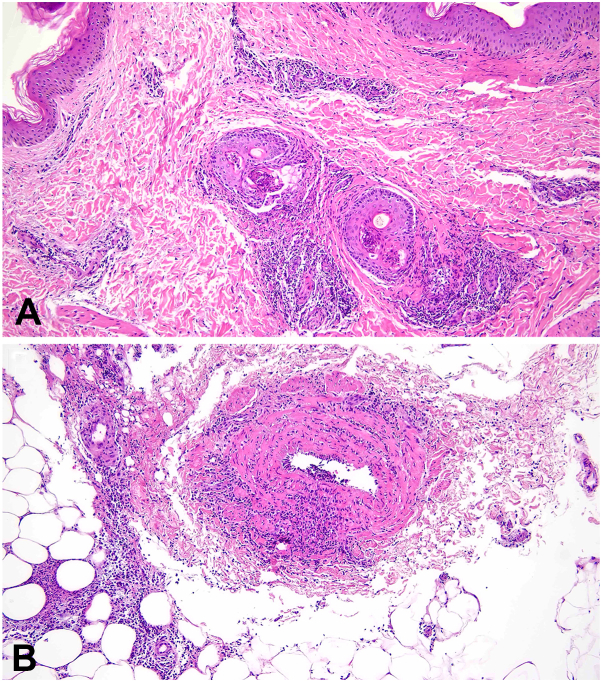
There is intense perivascular predominantly neutrophilic inflammation with invasion into and destruction of a small-to-medium sized vessel, shown at higher power at the dermal/subcutaneous fat junction (hematoxylin and eosin).

A punch biopsy from one of the right lower leg nodules demonstrated a number of histologic findings, including suppurative folliculitis, dermal lymphohistiocytic perivascular inflammation with scattered neutrophils, and lymphocytic vasculitis involving a medium-sized vessel with associated septal inflammation (Fig 2). Special stains for mycobacterial, bacterial, and fungal organisms were negative as was an immunostain for spirochetes. The biopsy features, while not entirely specific, in combination with the patient’s recurrent oral ulcers, new genital ulcer, pericarditis, and positive HLA-B∗51, supported a diagnosis of BD.^1^ The patient was treated with prednisone through delivery with subsequent resolution of her chest pain, nodules, and aphthae. At the time of this writing, the patient is 2 months postpartum.

## Discussion

While literature has described the effect of BD on subsequent pregnancy, the relationship between the 2 appears variable. Our case represents a rare onset of BD during a repeat pregnancy.

Fetal implantation results in a tolerogenic shift in immunoregulation from a T helper (Th)1/Th17 cell response to a Th2-/Treg-mediated response. Both Th1 and Th17 cells release pro-inflammatory cytokines, such as IL-2, IFN-γ, TNF-α, IL-17, IL-6, and IL-8, some of which are implicated in the pathogenesis of BD secondary to neutrophilic hyperactivation.^2^ Since pregnancy is associated with a protective effect against Th1-/Th17-mediated autoimmune diseases, it is particularly unusual that our patient first presented in her third trimester during an immunologically privileged period. However, estrogen is associated with the development of cutaneous lesions in early pregnancy such as erythema nodosum, which may have overlapping features with BD.^3^ Progesterone, which increases vascular permeability and promotes angiogenesis in early pregnancy, is associated with gingival hyperemia, oropharyngeal ulceration, and bleeding. Other conditions that have common overlapping presentation with BD include sarcoidosis, Crohn’s disease, and granulomatosis with polyangiitis.^4^

Recent studies support remission rates of 52% to 70% in pregnancy, suggesting disease quiescence during this period for most patients.^5^ Exacerbations most often present as recurrent oral and genital ulcers, as in our patient. Although BD tends to abate during pregnancy, the disease course varies even among different pregnancies in the same patient.^6^ Postpartum mothers often experience increased incidence of BD flare-ups, likely due to the restoration of baseline Th1/Th2 immunoregulation. Maternal-fetal complications such as preeclampsia have been reported very rarely. Several studies have suggested increased rates of preterm labor and cesarean section, although a consensus has not been established.^7^

To manage BD exacerbations during pregnancy and lactation, first-line therapies include corticosteroids and TNF-α inhibitors.^8^ Colchicine is commonly used as a second-line agent. Emerging therapies such as anakinra, secukinumab, and ustekinumab have shown promising results in cases refractory to conventional treatments based on open-label and retrospective observational data. Among treatment options for BD in pregnancy and breastfeeding, selection of an appropriate agent for recalcitrant disease should be guided by the specific organ involvement. Ustekinumab and secukinumab are particularly effective for mucocutaneous manifestations, while cyclosporine is recommended for ocular and vascular involvement, though contraindicated in neuro-BD.^9^ Rituximab is reserved for neuro-BD.^10^ While oral contraceptives may be taken prior to conception, peripartum use is contraindicated due to hypercoagulability. Vigilant postpartum care and thoughtful medication management are imperative to safeguard the health of both mother and child in patients with BD.

## Conflicts of interest

None disclosed.
